# Identification of Soybean Seed Varieties Based on Hyperspectral Imaging Technology

**DOI:** 10.3390/s19235225

**Published:** 2019-11-28

**Authors:** Shaolong Zhu, Maoni Chao, Jinyu Zhang, Xinjuan Xu, Puwen Song, Jinlong Zhang, Zhongwen Huang

**Affiliations:** School of Life Science and Technology, Henan Institute of Science and Technology/Collaborative Innovation Center of Modern Biological Breeding of Henan Province, Xinxiang 453003, China; zsl_94121@126.com (S.Z.);

**Keywords:** soybean seed, hyperspectral image, variety identification, combination model

## Abstract

Hyperspectral imaging is a nondestructive testing technology that integrates spectroscopy and iconology technologies, which enables us to quickly obtain both internal and external information of objects and identify crop seed varieties. First, the hyperspectral images of ten soybean seed varieties were collected and the reflectance was obtained. Savitzky-Golay smoothing (SG), first derivative (FD), standard normal variate (SNV), fast Fourier transform (FFT), Hilbert transform (HT), and multiplicative scatter correction (MSC) spectral reflectance pretreatment methods were used. Then, the feature wavelengths and feature information of the pretreated spectral reflectance data were extracted using competitive adaptive reweighted sampling (CARS), the successive projections algorithm (SPA), and principal component analysis (PCA). Finally, 5 classifiers, Bayes, support vector machine (SVM), k-nearest neighbor (KNN), ensemble learning (EL), and artificial neural network (ANN), were used to identify seed varieties. The results showed that MSC-CARS-EL had the highest accuracy among the 90 combinations, with training set, test set, and 5-fold cross-validation accuracies of 100%, 100%, and 99.8%, respectively. Moreover, the contribution of spectral pretreatment to discrimination accuracy was higher than those of feature extraction and classifier selection. Pretreatment methods determined the range of the identification accuracy, feature-selective methods and classifiers only changed within this range. The experimental results provide a good reference for the identification of other crop seed varieties.

## 1. Introduction

Seed varieties are directly related to the yield and quality of soybeans. Mixed and adulterated soybeans cause substantial problems for farmers and lead to seed market complexities [1]. At the same time, as people’s requirements for food quality become increasingly higher, it is necessary to process different uses according to different seed varieties. For example, the soymilk and tofu made by high-protein soybeans are more delicious [2,3]. Therefore, the identification of seed varieties is an urgent problem to be solved in agricultural production, seed sales, and food processing. The common methods of seed identification in China and abroad include morphological methods, the gel electrophoresis of soluble seed proteins [4,5], direct analysis with real-time mass spectrometry [6], isoenzyme electrophoresis [7], liquid chromatography [8], and simple sequence repeat (SSR) analysis [9]. Morphological methods are highly demanding of experience for appraisers and the identification accuracy is easy subject to subjective factors. The other methods mentioned above have high identification accuracy but are all destructive tests, and random sampling cannot achieve separation. Moreover, these methods have a long detection period and consume large amounts of chemical reagents, which causes chemical contamination; thus, these methods are not suitable for large-scale sample detection [10].

Spectral detection technology has the characteristics of fast detection speed, high efficiency, no pollution, and nondestruction. Spectral imaging technology is a form of remote sensing technology, in which each pixel in an image contains a large amount of spectral information, which can be used to detect the state of the object [11]. In agricultural production, this technology is mainly used to detect crop growth information, such as the contents of nitrogen, phosphorus, potassium, and chlorophyll [12,13]; spectral imaging is also used in crop maturity and yield prediction [14], drought disaster monitoring [15], disease and insect pest detection [16], soil nutrition analysis [17], and other analyses. In terms of grain quality and safety, spectral imaging technology is mainly used in seed viability and germination detection [18,19,20,21], seed composition detection [22], seed impurity detection [23], and pesticide residue detection [24]. Although spectral detection technology is widely used, it also has some difficulties. (1) The detection accuracy is very high when the internal structure of the object is simple, similar to the consistent forms of inorganic matter, such as petroleum [25] and minerals [26]; however, for organic substances (such as seeds) with complex and unevenly distributed internal chemical components, it is difficult to detect substantial differences among varieties. (2) Spectra contain a high amount of information, but they also contain a high amount of interference information, such as redundancy and noise, which further increases the difficulty of identification.

Spectrum pretreatment, feature extraction, and a classifier with good performance can solve the above problems to a certain extent. Li [27] obtained the reflective spectral information of melons with multiplicative scattering correction (MSC), standard normal variable (SNV) transform, first derivative (FD) analysis, and Savitzky-Golay smoothing (SG); used principal component analysis (PCA) to extract principal components; and established a Fisher discriminant model and distance discriminant model. The results showed that their discriminant accuracies were all higher than 90.0%. Although the identification of seed varieties for some crops has been published, such as for soybean [28], wheat [29,30], melon [31], oat [32], corn [33], and rice [34], there are many problems that need to be improved. Zhu [35] used near-infrared technology to identify soybean varieties with an identification accuracy rate of 100%, but there were only two soybean seed varieties: KenjianDou 43 was a variety with high isoflavone and high fat, while Zhonghuang 13 was a variety with high protein and low fat. Such material selection could not fully test the identification effect of the model. Tan [36] used the hyperspectral image technique in conjunction with the BP neural network to classify six soybean seed varieties, but the influences of the instrument, the sample itself, the environment, and other factors may cause a high amount of interference information, and this method does not take these factors into account. Feng [32] preprocessed near-infrared spectra using wavelet transform and SNV, extracted features with PCA and independent component analysis (ICA), and established support vector machine (SVM), k-nearest neighbor (KNN), and radial basis function neural network (RBFNN) models used for raisin variety identification. However, except when less material is used, PCA and ICA will transform the raw data, which is a type of data compression. The model effect will be more accurate and robust based on feature variable selection. In general, these problems are summarized as follows. (1) The number of varieties or samples selected is small, or materials with widely varying quality are deliberately chosen [34,37,38]. (2) The pretreatment and feature extraction are not given enough attention or not even pretreated [31,38,39]. (3) Although dozens of methods have been proposed in previous studies, pretreatment methods, feature extraction methods, and classifiers need to be combined to achieve optimal performance. Therefore, determining which three features comprise the best combination in the identification of soybean seed varieties is urgently needed, and there are no studies that have developed more systematic summaries.

To solve the above problems, in this paper, the hyperspectral images of 1200 soybeans of 10 soybean varieties were obtained, and 6 different pretreatments were applied to the seed spectral information. Three feature extraction methods and five classifiers were used to classify and identify the soybean seed varieties. The aim of this study was to determine the best combination of pretreatment methods, feature extraction methods, and classifiers, and provide technical support for establishing a rapid, accurate, nondestructive, and stable hyperspectral identification system for soybean varieties.

## 2. Materials and Methods

### 2.1. Materials

**Figure 1:**
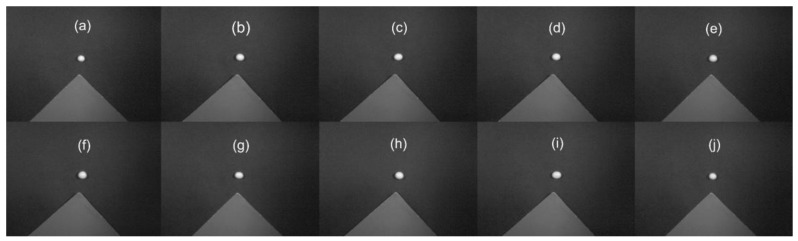
Hyperspectral images of each variety at 685 nm. (**a**) NanNong 1606, (**b**) ShangDou 161, (**c**) ShangDou 1201, (**d**) ShangDou 1310, (**e**) YuDou 18, (**f**) YuDou 22, (**g**) YuDou 25, (**h**) Zheng 196, (**i**) Zheng 3074, (**j**) Zheng 9525.

A total of 1200 soybeans from 10 varieties widely planted in the Huang-Huai-Hai Plain were collected as samples, and all samples came from the experimental field of the Henan Institute of Science and Technology. A total of 3 replicates were established in the experimental field, and 40 soybean seeds were selected for each repetition and each variety. All of the varieties have yellow seed coats, and the seeds were required to be whole and free from damage and disease spots (Figure 1). The crude protein and crude fat content of each variety are shown in Table 1.

### 2.2. Instruments and Hyperspectral Acquisition

Hyperspectral imaging systems include 4 parts: an imaging spectrometer, a light source, accessories, and analytical software. The hyperspectral imager (SOC 710VP, Surface Optics Corporation, America) has a built-in dual charge-coupled device (CCD) detector and pushbroom translation device with high integration, a spectral range of 373–1043 nm, a spectral resolution of 4.6875 nm, and a total of 128 bands. Two 100 W halogen lamps are used as the light source. In addition, the system is primarily composed of a standard gray Spectralon panel, darkroom, computer, and other accessories.

**Figure 2:**
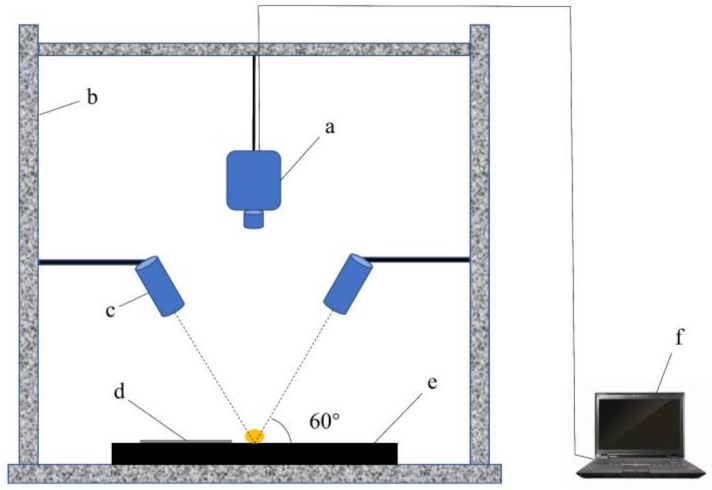
Hyperspectral imaging system. (**a**) Imaging spectrometer, (**b**) Darkroom, (**c**) Light source, (**d**) Standard gray Spectralon panel, (**e**) Loading stage, (**f**) Computer.

SOC710 Acquisition Software was used to collect the images, and before image acquisition, all samples were placed in an oven at 38 °C for 24 h. The standard gray Spectralon panel was placed directly below the seed, the spectrometer lens was 30 cm away from the stage, and the two light sources were placed on either side of the spectrometer with the incident light at an angle of 60° to the stage (Figure 2). The integration was set to 20 ms, and the gain was 3.

### 2.3. Image Correction and Reflectance Conversion

Dark current correction and radiation calibration of the collected images were conducted using SRAnal 710 software (radiation calibration files were provided by the spectrometer manufacturer) and the images were converted into float files that were read by ENVI software. Then, the image segmentation algorithm was used to obtain the complete seed image as the region of interest (ROI), and the average value of this region was taken as the spectral reflectivity. To solve the problem of the uneven distribution of radiance from the artificial light source, one soybean was placed flat on a black stage in the same position, and the reflectivities of the front and back sides of the bean were measured. The average of the two sides was taken as the spectral reflectivity of the soybean. The soybean was calculated by using the following equation:

(1) $$R=\frac{DN}{DN_{N}}\times R_{N}$$

where *R* is the reflectance of the soybean, DN is the digital number of the soybean, $DN_{N}$ and $R_{N}$ are, respectively, the digital number and reflectance of standard gray Spectralon panel. $R_{N}$ was obtained by precalibration in the laboratory. DN and $DN_{N}$ were measured in this experiment.

### 2.4. Pretreatment, Feature Extraction, and Classifier Selection

The influences of the instrument, the sample itself, the environment, and other factors may cause a high amount of interference, and preprocessing can remove the effects of noise, baseline drift, and scattering [40]. This study used pretreatment methods including SG, FD, SNV, fast Fourier transform (FFT), Hilbert transform (HT), MSC. The basic idea of SG is to fit the data in the moving window by polynomial least squares to achieve smoothing; the polynomial order is set to 2. The number of window points is set to 8, and the larger the value is, the smoother the spectral curve, but this method removes some important useful information. The derivative is also called the rate of change, and the derivative of waveband X is calculated as follows:

(2) $$FD_{\lambda \left(X\right)}=\frac{R_{\lambda \left(X+1\right)}-R_{\lambda \left(X-1\right)}}{\lambda_{\left(X+1\right)}-\lambda_{\left(X-1\right)}}$$

where $R_{\lambda \left(X+1\right)}$ is the reflectance at the next waveband of X, $R_{\lambda \left(X-1\right)}$ is the reflectance at the last waveband of X, $\lambda_{\left(X+1\right)}$ is the wavelength of the next waveband of X, and $\lambda_{\left(X-1\right)}$ is the wavelength of the last waveband of X.

The SNV and MSC methods were used to eliminate the scattering effect caused by an uneven particle distribution on the sample surface, and the SNV formula was:

(3) $$X_{SNV}=\frac{X-\bar{X}}{\sqrt{\frac{\sum_{i=1}^{p}\left(X_{i}-\bar{X}\right)}{p-1}}}$$

where X is the original spectrum of a sample; $\bar{X}$ is the spectral average of all the wavelength points in the sample; and i = 1, 2, …, p, p is the number of wavelength points.

For MSC, first, the average spectrum of the sample is calculated ($\bar{X}$). For a sample spectrum x, linear regression is performed between x and $\bar{X}$, $x=\alpha \bar{X}+\beta$, then the values of α and β are determined:

(4) $$x_{MSC}=\frac{x-\beta }{\alpha }.$$

By adjusting the values of α and β, the spectral difference can be reduced while retaining useful information in the original spectrum as much as possible.

FFT can smooth, filter, and convolve the original spectrum. In the spectral signal, the noise signal is generally considered a high-frequency signal. This experiment used FFT filtering with a low pass filter and a cutoff frequency of 0.125. HT is often used in signal processing and fault diagnosis in engineering applications. In a sense, HT is equivalent to a special filter.

Spectral data contain a high amount of redundant information and multiple collinearity problems that greatly affect the modeling speed and may even affect the model results. At present, the frequently used feature extraction methods include PCA [41,42,43], x-loading weight [44], competitive adaptive reweighted sampling (CARS) [42], wavelet transform [45,46], and the Kolmogorov-Smirnov test [47,48]. Among these methods, CARS, successive projections algorithm (SPA), and PCA are the most widely used and perform the best in most studies, this study compared these three methods.

The classifiers used in this study were Bayes, SVM, KNN, ensemble learning (EL), and artificial neural network (ANN), and the corresponding parameters are shown in Table 2. For each soybean variety, the spectra were randomly divided into a training set and test set at a 3:1 ratio, and the validation method was 5-fold cross-validation. All processing was completed by MATLAB R2019a (MathWorks, USA).

## 3. Results and Discussion

### 3.1. Hyperspectral Characteristics

**Figure 3:**
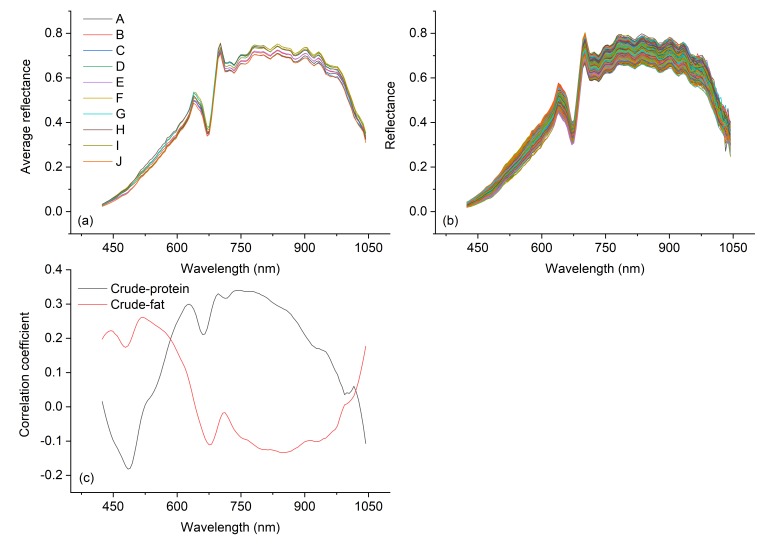
Soybean seed spectral curves and correlation coefficients. (**a**) Average reflectivity of each soybean variety. A: NanNong 1606; B: ShangDou 161; C: ShangDou 1201; D: ShangDou 1310; E: YuDou 18; F: YuDou 22; G: YuDou 25; H: Zheng 196; I: Zheng 3074; J: Zheng 9525. (**b**) Reflectivity of all samples. (**c**) Correlations of spectral reflectivity with crude protein and crude fat.

Figure 3a,b shows that the trends of the hyperspectral curves of different varieties of soybean seeds are very similar: there are peaks at 638 nm and 702 nm and a valley at 675 nm, and all three occur in the range of red light (620–750 nm). Compared with other bands, the spectra of each variety at 638–660 nm and 700–980 nm are significantly different. The correlation coefficients were determined by comparing the spectral reflectivity of each band with the crude protein content and crude fat content (Figure 3c), and the highest value was 0.34 at 749 nm, indicating that the spectral reflectance of different varieties is not significantly related to the crude protein content or crude fat content.

### 3.2. Pretreatment Analysis

**Figure 4:**
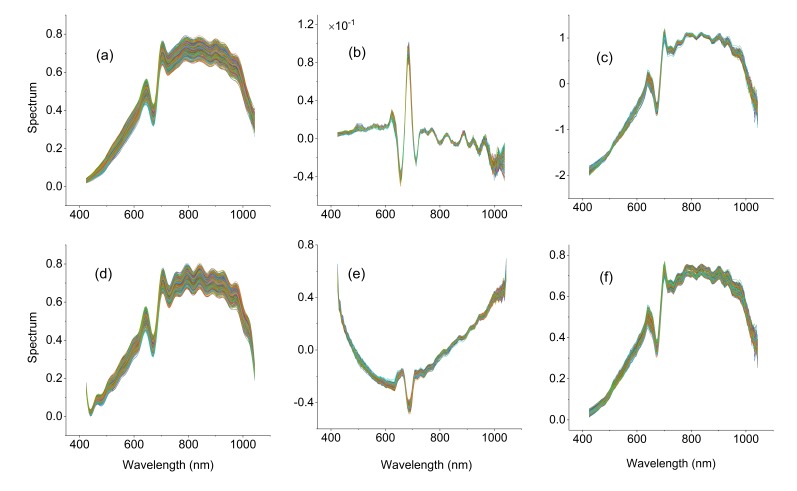
Spectrum curves of all samples preprocessed by Savitzky-Golay smoothing (SG), first derivative (FD), standard normal variate (SNV), fast Fourier transform (FFT), Hilbert transform (HT), and multiplicative scatter correction (MSC). (**a**) SG, (**b**) FD, (**c**) SNV, (**d**) FFT, (**e**) HT, and (**f**) MSC.

The pretreatment results are shown in Figure 4. The SG (Figure 4a) and FFT (Figure 4d) results are smoother than the results of Figure 3b and eliminate the noise of the original spectrum at 1000 nm. One of the common points of the two methods is fitting the low-frequency component in the signal and removing the high-frequency component. Since the two methods do not involve the average spectra of all samples, the difference between each spectral curve is still large. The geometric meaning of the derivative is the tangent slope of the curve at a certain point, so the derivative can magnify the difference. With FD pretreatment, the spectral differences among different soybeans are mainly in the ranges of 623–638 nm, 649–659 nm, and 675–687 nm (Figure 4b), and these different bands are all within the range of bands with large differences in the original spectra, which indicates that the derivative transformation highlights the characteristic wavelengths. According to the formulas of the SNV and MSC methods, both preprocessing methods need to be calculated based on the average spectrum of all samples. Therefore, the SNV (Figure 4c) and MSC (Figure 4f) results were significantly reduced between samples after pretreatment compared with the results in Figure 3b. The HT changes the frequency components by introducing a phase shift of −90 degrees at each positive frequency and a phase shift of 90 degrees at each negative frequency. The amplitudes are left unaltered. By applying an inverse Fourier transform on the product, we can obtain the HT of the input data. After HT preprocessing, there is still a high amount of noise (Figure 4e). HT is worse than common filtering methods.

### 3.3. Feature Extraction Analysis

#### 3.3.1. CARS

**Figure 5:**
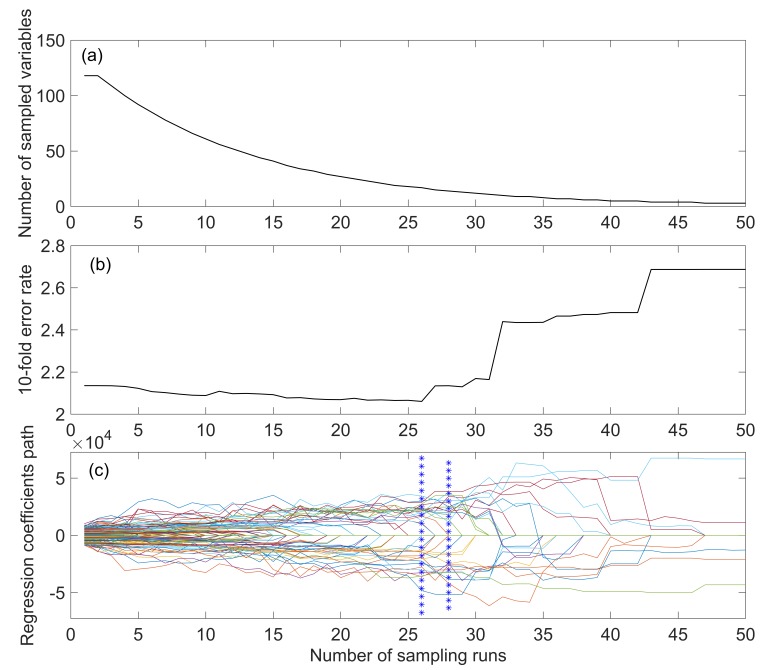
Curves showing the process of characteristic wavelength selection for SG pretreatment by competitive adaptive reweighted sampling (CARS). (**a**) Number of bands retained with the number of sampling runs. (**b**) Change curve of root mean square error of cross-validation (RMSECV) with the number of sampling runs. (**c**) Regression coefficient paths with the number of sampling runs.

Figure 5 shows the characteristic wavelength screening process after SG smoothing. As the number of runs increases, the number of bands retained decreases rapidly first and then slowly (Figure 5a). At 0–29 sampling runs, the root mean square error of cross-validation (RMSECV) decreases slowly (Figure 5b), indicating that the eliminated bands have little influence on the RMSECV, but a sudden rise occurs after 29 sampling runs, which indicates that the key band has been removed and that important information has been lost, resulting in a large RMSECV value. The positions marked by ‘*’ in Figure 5c show where the RMSECV reaches a minimum at the bands retained in the 8th and 23rd sampling runs. Twenty-one retained bands were screened by CARS: 495 nm, 505 nm, 515 nm, 520 nm, 525 nm, 597 nm, 665 nm, 691 nm, 707 nm, 718 nm, 723 nm, 739 nm, 750 nm, 755 nm, 787 nm, 814 nm, 819 nm, 846 nm, 895 nm, 911 nm, and 988 nm. Similarly, CARS was used to screen the characteristic wavelengths of the five other pretreatments, and the extracted band numbers were 21, 72, 41, 23, 25, and 78.

#### 3.3.2. SPA

**Figure 6:**
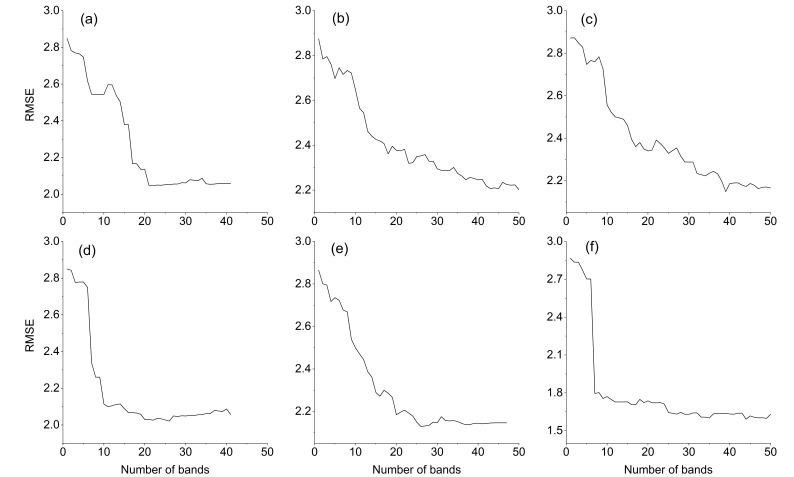
RMSE curves with the number of selected bands. (**a**) SG pretreatment. (**b**) FD pretreatment. (**c**) SNV pretreatment. (**d**) FFT pretreatment. (**e**) HT pretreatment. (**f**) MSC pretreatment.

The RMSE is large when the number of selected bands is small (Figure 6); then, as the number of selected bands increases, the RMSE decreases. However, after the number of bands reaches a certain threshold, the RMSE remains almost unchanged. Therefore, the extracted band numbers are 19, 42, 32, 10, 20, and 27.

#### 3.3.3. PCA

**Figure 7:**
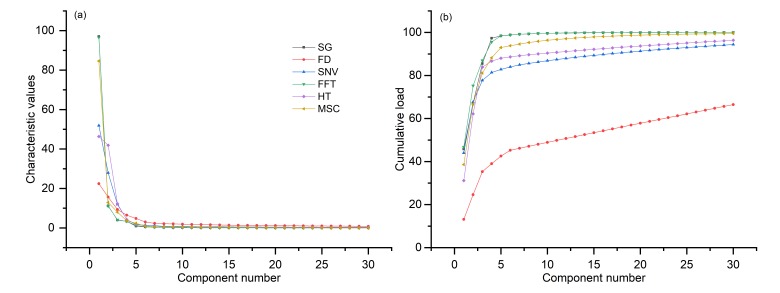
(**a**) Characteristic values of the first 30 principal components. (**b**) Cumulative loads of the first 30 principal components.

Through PCA, the characteristic values of the first 30 components were determined in this study (Figure 7a). A characteristic value less than 1 indicates that the principal component is not as powerful as the direct use of the original variable. Therefore, principal components with eigenvalues greater than 1 were screened. The numbers of principal component factors extracted from the six pretreatments were 4, 24, 7, 5, 5, and 5, and the cumulative loads were 97.3%, 61.3%, 85.0%, 98.4%, 88.0%, and 93.0%, respectively (Figure 7b). In contrast, SG, SNV, FFT, HT, and MSC extracted very few principal components, but the cumulative loads were very high, as all reached a value of more than 84%. The cumulative load of 24 principal components extracted based on FD pretreatment was only 61.3%, which was the most serious loss compared with the other five pretreatments.

### 3.4. Comparison of Identification Models

**Figure 8:**
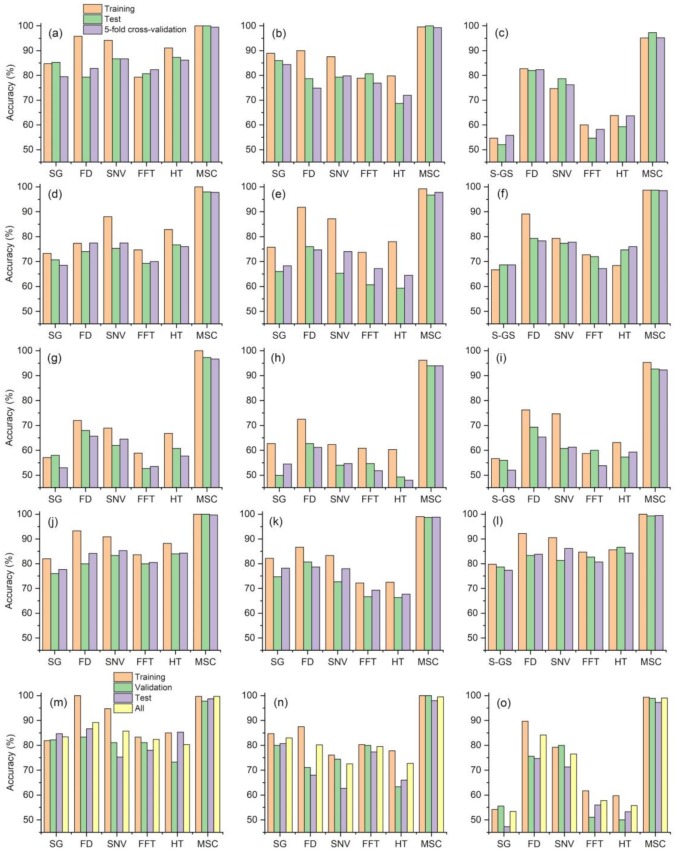
Accuracies of each combination. (**a**) CARS-Bayes, (**b**) SPA-Bayes, (**c**) PCA-Bayes, (**d**) CARS-SVM, (**e**) SPA-SVM, (**f**) PCA-SVM, (**g**) CARS-KNN, (**h**) SPA-KNN, (**i**) PCA-KNN, (**j**) CARS-EL, (**k**) SPA-EL, (**l**) PCA-EL, (**m**) CARS-ANN, (**n**) SPA-ANN, (**o**) PCA-ANN.

Six pretreatment methods, three feature extraction methods, five classifiers, and a total of 90 combinations were used in the identification of soybean seed varieties (Figure 8). The MSC-CARS-EL combination obtained the highest accuracy, reaching accuracies of 100%, 100%, and 99.8%. Of the six types of pretreatments, MSC performed best, and the training set, verification set, and 5-fold cross-validation accuracies of MSC were all above 92%. This result agrees with the work of other researchers [49,50]. However, in some other studies, MSC was not the optimal pretreatment method; rather, these studies reported optimal pretreatment methods of median filter smoothing [51], SG [52] t-distributed stochastic neighborhood embedding (t-SNE) [53], and SNV [54,55]. In the hyperspectral identification of wheat [56] and maize [57] seed varieties, the best models for identifying seed varieties were the 5 point and 3 time smoothing and SNV. At present, there is no literature proving that one pretreatment method is better than other pretreatment methods. In practical applications, different types of pretreatments need to be compared to determine the optimal method. To achieve improved pretreatment effects, two pretreatment methods can be used simultaneously. Research has indicated that [58] the use of SG combined with FD, SNV pretreatment combined with FD, and logarithmic transformation (LT) combined with FD increase the accuracy by approximately 15% relative to a single pretreatment. At present, pretreatment combination methods are rarely applied to the identification of seed varieties of cereals, and most of the combination methods use two types of pretreatments; the combination of 3 or more pretreatment methods is rare.

There were few differences in the three feature extraction methods, and CARS performed slightly better than SPA and PCA. CARS was indeed very accurate, but there may be better and more stable methods. In the future, more feature extraction methods can be studied, and the advantages and disadvantages of each method can be compared. Of the five classifiers, EL performed the best, and KNN performed the worst. Regarding the choice of classifiers, each method has applicable environments, conditions, and limitations [59]. For example, KNN requires a large sample size, and the discrimination accuracy is high when there are many overlapping samples to be classified; however, the identification accuracy for rare categories or imbalanced samples is poor, the required storage space is large, and the method requires a long computation time [60]. The most suitable discrimination method should be chosen according to the actual situation. Although the feature extraction method and classifier have less of an influence on accuracy than the pretreatment method, comprehensive method selection is an indispensable part of seed variety identification.

**Figure 9:**
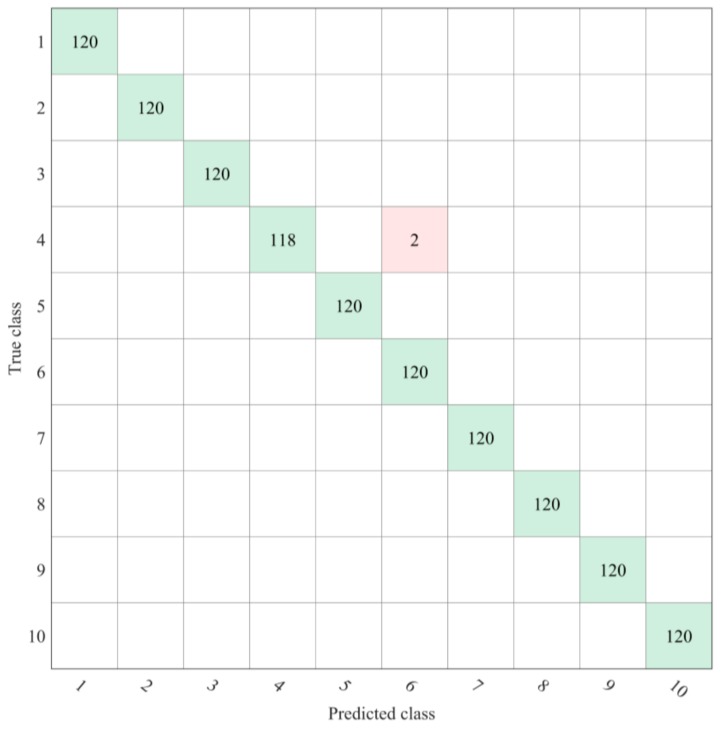
Confusion matrix of 5-fold cross-validation accuracy. 1: NanNong 1606. 2: ShangDou 161. 3: ShangDou 1201. 4: ShangDou 1310. 5: YuDou 18. 6: YuDou 22. 7: YuDou 25. 8: Zheng 196. 9: Zheng 3074. 10: Zheng 9525.

All of the combinations in this study misjudged the same 2 seeds of ShangDou 1310 beans as YuDou 22 beans (Figure 9). The reasons for this result are as follows: (1) Due to human factors, there were large errors in the process of collecting the images and selecting the ROI. (2) ShangDou 1310 was bred with YuDou 22 and Shang 8653-1-1-1-3-2; thus, ShangDou 1310 and YuDou 22 had many of the same gene sequences. In addition, due to environmental and other factors, the hyperspectral characteristics of the 2 seeds of ShangDou 1310 soybeans tended to be similar to those of YuDou 22. This experiment used a relatively low-resolution spectrometer, and a higher resolution and more accurate instruments may be required to achieve the accurate identification of these seed varieties.

At present, the identification of seed varieties based on spectral reflectance will not meet the needs of the public. The ‘graph-spectrum’ combination method is a future development direction and is better than individual methods [41,61,62]. Seed identification technology based on spectral reflectance has gradually developed, but image-based seed identification technology has not achieved satisfactory results. With the development of computer technology and information technology, the automatic identification of seed varieties and quality by machine vision combined with deep learning is an inevitable trend [20,63,64]. Moreover, hyperspectral technology is not limited to seed identification. The increasing resolution of spectrometers on unmanned aerial vehicles (UAVs) and satellites is one of the most important means to achieve precise and smart agricultural data. This technology can replace visual observations to monitor all aspects of agricultural production and has unlimited development potential.

## 4. Conclusions

In this study, we used six pretreatment methods, three feature extraction methods, and five classifiers, and a total of 90 combination models were comparatively analyzed to identify ten soybean seed varieties and determine the best model combination. The test results show that the MSC-CARS-EL model combination obtained the highest accuracy, and the selection of pretreatment methods had the greatest impact on the accuracy of the hyperspectral identification of soybean seed varieties. In a future study, we intend to select more soybean seed varieties to test the performance of this model combination, and we hope that the hyperspectral identification system of soybean seed varieties can be established for real-world applications.

## Figures and Tables

**Table 1:** Crude protein and crude fat content of each variety.

Variety	Crude Protein (%)	Crude Fat (%)	Variety	Crude Protein (%)	Crude Fat (%)
NanNong 1606	36.0	19.7	YuDou 22	46.5	18.9
ShangDou 161	35.6	19.6	YuDou 25	46.3	17.1
ShangDou 1201	43.1	20.2	Zheng 196	40.7	19.5
ShangDou 1310	42.1	20.5	Zheng 3074	40.9	17.1
YuDou 18	44.5	18.8	Zheng 9525	45.0	17.7

**Table 2:** Classifier parameters of each classifier. Bayes, SVM: Support vector machine, KNN: K-nearest neighbor, EL: Ensemble learning, ANN: Artificial neural network.

Classifiers	Parameters	Values
Bayes	Kernel	Gaussian
SVM	Kernel Function	Quadratic
	Box Constraint Level	1
	Multiclass Method	One-vs-One
KNN	Number of Neighbors	10
	Distance Metric	Euclidean
	Distance Weight	Equal
EL	Ensemble Method	Subspace
	Number of Learners	30
	Learning Rate	0.1
	Subspace Dimension	40
	Type of Neural Network	Back Propagation
ANN	Number of Hidden Neurons	10
	Training Function	Trainscg
